# Supplementary material for: Korean physicians’ perceptions regarding disclosure of patient safety incidents: A cross-sectional study
Source: PLoS One. 2020 Oct 8;15(10):e0240380. doi: 10.1371/journal.pone.0240380 (PMC7544042; doi:10.1371/journal.pone.0240380)
Supplement: S1 File — (DOCX) [file pone.0240380.s001.docx]

**Perceptions regarding Disclosure of Patient Safety Incidents Survey**

Department of Preventive Medicine, Ulsan University Hospital

| Dear participants.  The purpose of this survey is to understand the perceptions regarding disclosure of patient safety incident (DPSI), which is known to improve the standard of patient safety, of the general public and the medical professionals. The result of the survey will be used as a practical resource for the future development of the introduction and application of DPSI.  We will do our best to keep your information confidential and secured. The result of this study will be used for scholarly purposes only, and your personal information will be anonymized to prevent identification for the scholarly use also.  The survey will take about 10 minutes, and you will receive two complimentary coffee coupons if you leave your mobile number when you complete the survey.  Thank you for taking the time to cooperate and help our research.  October 2018 |
| --- |

| **Perceptions regarding Disclosure of Patient Safety Incidents** |
| --- |

| 1) DPSI according to the level of harm resulting from medical errors | Strongly disagree | Disagree | Agree | Strongly agree |
| --- | --- | --- | --- | --- |
| 1-1) Major errors should be disclosed to patients or their caregivers. | 1 | 2 | 3 | 4 |
| 1-2) Minor errors should be disclosed to patients or their caregivers. | 1 | 2 | 3 | 4 |
| 1-3) Near misses should be disclosed to patients or their caregivers. | 1 | 2 | 3 | 4 |

| 2) DPSI according to various scenarios in patient safety incidents | Strongly disagree | Disagree | Agree | Strongly agree |
| --- | --- | --- | --- | --- |
| 2-1) DPSI should be performed even in a physician thinks that patients and their caregivers would not be able to understand what the physician said. | 1 | 2 | 3 | 4 |
| 2-2) DPSI should be performed even if a physician thinks that patients and their caregivers would not want to know patient safety incidents. | 1 | 2 | 3 | 4 |
| 2-3) DPSI should be performed even if a physician thinks that patients and their caregivers could not know whether patient safety incidents occurred without being told. | 1 | 2 | 3 | 4 |
| 2-4) DPSI should be performed even if a physician thinks that patients and their caregivers have nothing to gain by having patient safety incidents acknowledged. | 1 | 2 | 3 | 4 |
| 2-5) The better the previous physician-patient relationship, the more DPSI will be performed. | 1 | 2 | 3 | 4 |

| 4) Barriers to DPSI | Strongly disagree | Disagree | Agree | Strongly agree |
| --- | --- | --- | --- | --- |
| 4-1) DPSI will increase the incidence of medical lawsuits. | 1 | 2 | 3 | 4 |
| 4-2) If DPSI is performed, a physician will lose his or her honor. | 1 | 2 | 3 | 4 |
| 4-3) if DPSI is performed, the physician will be punished by his or her hospital. | 1 | 2 | 3 | 4 |
| 4-4) A physician who performs DPSI is less competent. | 1 | 2 | 3 | 4 |
| 4-5) If DPSI is performed, the physician will be criticized by his or her colleagues. | 1 | 2 | 3 | 4 |
| 4-6) It is unreasonable to demand DPSI in only the medical field, and disclosure is not actively conducted in other fields. | 1 | 2 | 3 | 4 |

| **Facilitation of Disclosure of Patient Safety Incidents** |
| --- |

| 1) Methods for facilitating DPSI | Strongly disagree | Disagree | Agree | Strongly agree |
| --- | --- | --- | --- | --- |
| 1-1) It is necessary to strengthen the ethical mindset of physicians for DPSI. | 1 | 2 | 3 | 4 |
| 1-2) A training course for DPSI is needed. | 1 | 2 | 3 | 4 |
| 1-3) Manpower to support DPSI in hospitals is required. | 1 | 2 | 3 | 4 |
| 1-4) A guideline for DPSI is needed. | 1 | 2 | 3 | 4 |

| * To impose DPSI, some countries have enacted an apology law that discharges detrimentality of physicians for disclosing and apologizing their errors to patients. The apology law was first adopted by the state of Massachusetts in the United States in 1986, and as of 2009, 36 states have implemented the apology law. The apology law mainly states that an apology made by a physician is not regarded as an admission of a civil liability. Also, some states enacted a law that mandates DPSI. |
| --- |

| 2) Apology Methods | Strongly disagree | Disagree | Agree | Strongly agree |
| --- | --- | --- | --- | --- |
| 2-1) If apology law is enacted, physicians will perform more DPSI. | 1 | 2 | 3 | 4 |
| 2-2) Apology law will limit patients’ ability to prove physicians’ negligence. | 1 | 2 | 3 | 4 |
| 2-3) I support the introduction of apology law. | 1 | 2 | 3 | 4 |
| 2-4) I support the introduction of mandatory DPSI by law. | 1 | 2 | 3 | 4 |

| **Socio-demographic factor** |
| --- |

1. What is your sex?

① Male

② Female

2. In what age group are you?

① The 20s

② The 30s

③ The 40s

④ The 50s

⑤ Over the 60s

3. How many years have you practiced medicine? (On the basis of period after the license acquisition)

( ) years

4. If you write down your mobile number, we will assume that you have agreed to provide personal information and will provide two complimentary coffee coupons for participating in the survey. If you do not agree, do not fill out this form.

( )

The survey is completed.

Thank you for taking the time to complete this survey.
