# Supplementary material for: Korean physicians’ perceptions regarding disclosure of patient safety incidents: A cross-sectional study
Source: PLoS One. 2020 Oct 8;15(10):e0240380. doi: 10.1371/journal.pone.0240380 (PMC7544042; doi:10.1371/journal.pone.0240380)
Supplement: S2 File — (DOCX) [file pone.0240380.s002.docx]

**환자안전사건 소통하기 인식 등에 관한 설문조사**

울산대학교병원 예방의학과

| 안녕하십니까.  이번 설문조사에서는 우리나라의 환자안전 관련 문제에 대한 전반적인 인식과 더불어 환자안전의 수준을 높이는 것으로 알려진 환자안전사건 소통하기에 대하여 의료인들이 어떻게 생각하고 있는지 확인하고자 합니다. 이번 설문조사의 결과는 향후 환자안전사건 소통하기 도입 및 활용에 유용한 자료로 활용될 것입니다.  귀하의 개인정보가 유출되지 않도록 만전을 기하도록 하겠습니다. 이번 연구의 결과는 학술적 목적 이외에는 사용되지 않을 것이고, 학술적 목적으로 사용하는 경우에도 개인정보를 확인할 수 없도록 익명화할 것입니다.  설문조사는 약 10분 정도 소요되고, 설문을 모두 완료하신 분 중 휴대폰 번호를 남겨주신 분들에게는 커피 쿠폰 2매를 제공해드릴 예정입니다.  바쁘시더라도 잠시만 시간을 내어 협조해 주시면 큰 도움이 되겠습니다.  2018년 10월 |
| --- |

| **환자안전사건 소통하기 인식** |
| --- |

| 1) 의료오류의 심각성에 따른 환자안전사건 소통하기 | 전혀 동의하지 않음 | 동의  하지 않음 | 동의함 | 매우  동의함 |
| --- | --- | --- | --- | --- |
| 1-1) 심각한 의료오류가 발생한 경우 의사는 이 사실을 환자 및 보호자에게 알려야 한다. | 1 | 2 | 3 | 4 |
| 1-2) 가벼운 의료오류가 발생한 경우 의사는 이 사실을 환자 및 보호자에게 알려야 한다. | 1 | 2 | 3 | 4 |
| 1-3) 환자에게 위해가 가해지지 않은 오류가 발생한 경우 의사는 이 사실을 환자 및 보호자에게 알려야 한다. | 1 | 2 | 3 | 4 |

| 2) 관련 상황에 따른 환자안전사건 소통하기 | 전혀 동의하지 않음 | 동의  하지 않음 | 동의함 | 매우  동의함 |
| --- | --- | --- | --- | --- |
| 2-1) 환자 및 보호자가 설명을 잘 이해하지 못할 것이라고 의사가 판단한 경우에도 환자안전사건 소통하기를 해야 된다. | 1 | 2 | 3 | 4 |
| 2-2) 환자 및 보호자가 환자안전사건을 알려고 하지 않을 것이라고 의사가 판단한 경우에도 환자안전사건 소통하기를 해야 된다. | 1 | 2 | 3 | 4 |
| 2-3) 환자 및 보호자가 환자안전사건 발생을 알기가 힘들 것이라고 의사가 판단한 경우에도 환자안전사건 소통하기를 해야 된다. | 1 | 2 | 3 | 4 |
| 2-4) 환자 및 보호자가 환자안전사건 발생을 알게 되면 환자 및 보호자에게 득이 될 것이 없다고 의사가 판단한 경우에도 환자안전사건 소통하기를 해야 한다. | 1 | 2 | 3 | 4 |
| 2-5) 의사와 환자 간 이전 관계가 더 좋을수록 의사는 더 환자안전사건 소통하기를 할 것이다. | 1 | 2 | 3 | 4 |

| 3) 환자안전사건 소통하기의 효과 | 전혀 동의하지 않음 | 동의  하지 않음 | 동의함 | 매우  동의함 |
| --- | --- | --- | --- | --- |
| 3-1) 환자안전사건 소통하기를 하면 환자 및 보호자는 의사를 더 신뢰할 것이다. | 1 | 2 | 3 | 4 |
| 3-2) 환자안전사건 소통하기를 하는 의사를 주변 사람에게 더 추천할 것이다. | 1 | 2 | 3 | 4 |
| 3-3) 환자안전사건 소통하기를 하는 의사에게 다시 진료를 받을 것이다. | 1 | 2 | 3 | 4 |
| 3-4) 환자안전사건 소통하기를 하는 의사가 더 좋은 의료를 제공할 것이다. | 1 | 2 | 3 | 4 |
| 3-5) 환자안전사건 소통하기를 하면 본인 스스로 환자안전 문제에 관심을 더 가질 것이다, | 1 | 2 | 3 | 4 |
| 3-6) 환자안전사건 소통하기를 하면 의사의 죄의식이 감소할 것이다. | 1 | 2 | 3 | 4 |

| 4) 환자안전사건 소통하기의 장애물 | 전혀 동의하지 않음 | 동의  하지 않음 | 동의함 | 매우  동의함 |
| --- | --- | --- | --- | --- |
| 4-1) 환자안전사건 소통하기를 하면 의료소송이 증가할 것이다. | 1 | 2 | 3 | 4 |
| 4-2) 환자안전사건 소통하기를 하면 의사의 명예가 실추될 것이다. | 1 | 2 | 3 | 4 |
| 4-3) 환자안전사건 소통하기를 하면 의사는 의료기관의 징계를 받게 될 것이다. | 1 | 2 | 3 | 4 |
| 4-4) 환자안전사건 소통하기를 하는 의사가 더 실력 없는 의사이다. | 1 | 2 | 3 | 4 |
| 4-5) 환자안전사건 소통하기를 하면 의사는 동료들의 비난을 받게 될 것이다. | 1 | 2 | 3 | 4 |
| 4-6) 의료계에서만 환자안전사건 소통하기를 요구하는 것은 무리이다. | 1 | 2 | 3 | 4 |

| **환자안전사건 소통하기 촉진** |
| --- |

| 1) 환자안전사건 소통하기 촉진 방법 | 전혀 동의하지 않음 | 동의  하지 않음 | 동의함 | 매우  동의함 |
| --- | --- | --- | --- | --- |
| 1-1) 환자안전사건 소통하기를 위해서는 의사의 윤리 의식을 높일 필요가 있다. | 1 | 2 | 3 | 4 |
| 1-2) 환자안전사건 소통하기를 교육하는 과정이 필요하다. | 1 | 2 | 3 | 4 |
| 1-3) 환자안전사건 소통하기를 지원하는 의료기관 내 인력이 필요하다. | 1 | 2 | 3 | 4 |
| 1-4) 환자안전사건 소통하기를 위한 가이드라인 마련이 필요하다. | 1 | 2 | 3 | 4 |

| * 환자안전사건 소통하기를 위하여 몇몇 국가들에서는 의료인들이 그들의 실수를 환자에게 공개하고 사과하는 것에 대한 불이익을 없애주기 위한 사과법(apology law)을 도입하였다. 1986년 미국의 매사추세츠(Massachusetts) 주에서 사과법을 처음 채택한 후, 2009년 기준 미국의 36개 주에서 사과법을 시행하고 있다. 사과법의 주 내용은 의료인의 사과를 민사적 법적 책임(civil liability)에 대한 시인(admission)으로 간주하지 않는다는 것이다. 나아가 일부 주에서는 환자안전사건 소통하기를 강제하는 법도 두고 있다. |
| --- |

| 2) 사과법 | 전혀 동의하지 않음 | 동의  하지 않음 | 동의함 | 매우  동의함 |
| --- | --- | --- | --- | --- |
| 2-1) 사과법을 제정하면 의사가 좀 더 환자안전사건 소통하기를 하게 될 것이다. | 1 | 2 | 3 | 4 |
| 2-2) 사과법은 의료소송에서 환자의 입증 능력을 감소시킬 것이다. | 1 | 2 | 3 | 4 |
| 2-3) 우리나라에서도 사과법을 제정하는 것을 찬성한다. | 1 | 2 | 3 | 4 |
| 2-4) 우리나라에서도 환자안전사건 소통하기를 강제하는 법을 제정하는 것을 찬성한다. | 1 | 2 | 3 | 4 |

| **인구사회학적 질문** |
| --- |

1. 귀하의 성별은 무엇입니까?

① 남성

② 여성

2. 귀하의 연령대는 어떻게 되십니까? (만 연령 기준)

① 20대

② 30대

③ 40대

④ 50대

⑤ 60대 이상

3. 귀하의 경력은 어떻게 되십니까? (면허증 취득 후 기간 기준)

( )년

4. 귀하의 휴대폰 번호를 적어주시면 개인정보제공에 동의하신 것으로 간주하여 설문참여 답례품(커피 쿠폰 2매)을 제공해드릴 예정입니다. 동의하지 않으시면 기입하지 않으셔도 됩니다.

( )

이상으로 설문조사가 끝났습니다.

끝까지 조사에 응해주셔서 감사합니다.
