# Supplementary material for: Korean physicians’ perceptions regarding disclosure of patient safety incidents: A cross-sectional study
Source: PLoS One. 2020 Oct 8;15(10):e0240380. doi: 10.1371/journal.pone.0240380 (PMC7544042; doi:10.1371/journal.pone.0240380)
Supplement: Attachment — Submitted filename: Comments to the Author.pdf [file pone.0240380.s003.pdf]

Comments to the Author

Thank you for the opportunity to review this article, titled "Korean physicians' perceptions regarding disclosure of patient safety incidents: A cross-sectional study."

My suggestions for revision are:

Although the title of the paper says "Korean physicians' perceptions regarding disclosure of patient safety incidents: A cross-sectional study," the author emphasized the gap between physicians' perceptions and the general public's perceptions from the introduction to the conclusion of the paper. Although the difference between physicians' perceptions and general public's perceptions was well presented through comparison, the discussion seems to be mainly concentrated on improvements and suggestions on the side of physicians. While physicians' perceptions should be changed and legal and institutional support for change is necessary, the approach on the side of the general public also seems to be important, but there seems to be a lack of discussion of it. In particular, although measures to narrow the gap between physicians' perceptions and general public's perceptions of barriers to DPSI are important, it would be good to consider points to be discussed on the side of the general public for improvement of the areas perceived as barriers by physicians and the general public.

In addition, the researcher emphasized the necessity of repeated studies with expanded study subjects (applying various countries, institutions, occupations, etc.). If the author has a concern about areas that could not be measured or areas that should be corrected or supplemented in the questionnaire applied in this study, it may be good to add them to the limitations.

Thank you for your writing a good paper.
